# A genetically engineered *Escherichia coli* strain overexpressing the nitroreductase NfsB is capable of producing the herbicide D-DIBOA with 100% molar yield

**DOI:** 10.1186/s12934-019-1135-8

**Published:** 2019-05-20

**Authors:** Maria Elena de la Calle, Gema Cabrera, Domingo Cantero, Antonio Valle, Jorge Bolivar

**Affiliations:** 1 Department of Chemical Engineering and Food Technology, University of Cadiz, Campus Universitario de Puerto Real, Puerto Real, 11510 Cadiz, Spain; 2 Department of Biomedicine, Biotechnology and Public Health-Biochemistry and Molecular Biology, University of Cadiz, Campus Universitario de Puerto Real, Puerto Real, 11510 Cadiz, Spain; 3 Institute of Viticulture and Agri-Food Research (IVAGRO)-International Campus of Excellence (ceiA3), University of Cadiz, Puerto Real, Spain; 4 Institute of Biomolecules (INBIO), University of Cadiz, Puerto Real, Spain

**Keywords:** Allelochemical herbicides, D-DIBOA, *E. coli*, Nitroreductases, NfsB, Genetic modification, Whole-cell-biocatalyst

## Abstract

**Background:**

The use of chemical herbicides has helped to improve agricultural production, although its intensive use has led to environmental damages. Plant allelochemicals are interesting alternatives due to their diversity and degradability in the environment. However, the main drawback of this option is their low natural production, which could be overcome by its chemical synthesis. In the case of the allelochemical DIBOA ((2,4-dihydroxy-2H)-1,4-benzoxazin-3(4H)-one), the synthesis of the analogous compound D-DIBOA (2-deoxy-DIBOA) has been achieved in two steps. However, the scale up of this synthesis is hindered by the second step, which uses an expensive catalyst and is an exothermic reaction, with hydrogen release and a relatively low molar yield (70%). We have previously explored the “Green Chemistry” alternative of using *E. coli* strains overexpressing the nitroreductase NfsB as a whole-cell-biocatalyst to replace this second step, although the molar yield in this case was lower than that of the chemical synthesis.

**Results:**

In this work, we engineered an *E. coli* strain capable of carrying out this reaction with 100% molar yield and reaching a D-DIBOA concentration up to 379% respect to the highest biotransformation yield previously reported. This was achieved by a screening of 34 *E. coli* mutant strains in order to improve D-DIBOA production that led to the construction of the Δ*lapA*Δ*fliQ* double mutant as an optimum genetic background for overexpression of the NfsB enzyme and D-DIBOA synthesis. Also, the use of a defined medium instead of a complex one, the optimization of the culture conditions and the development of processes with several substrate loads allowed obtaining maxima yields and concentrations.

**Conclusions:**

The high yields and concentrations of D-DIBOA reached by the microbial-cell-factory approach developed in this work will facilitate its application to industrial scale. Also, the use of an optimized defined medium with only an organic molecule (glucose as carbon and energy source) in its composition will also facilitate the downstream processes.

**Electronic supplementary material:**

The online version of this article (10.1186/s12934-019-1135-8) contains supplementary material, which is available to authorized users.

## Background

One of the key elements to improve agricultural production is to eliminate the negative impact of weeds on the growth and quality of commercial crops [1]. The use of chemical herbicides has helped to mitigate this problem [2], although its intensive use has led to weed resistance and their chemical stability has caused environmental damages [3–5]. One of the most interesting alternatives to the chemical herbicides is the use of plant allelochemicals, which are natural products that affects the survival or growth of other organisms (plants, insects, microorganisms, etc.…) [6, 7]. Due to their chemical structure diversity, specific mode of action and degradability in the environment, these compounds have been considered new effective tools for agricultural pest management [5, 8].

**Fig. 1:**
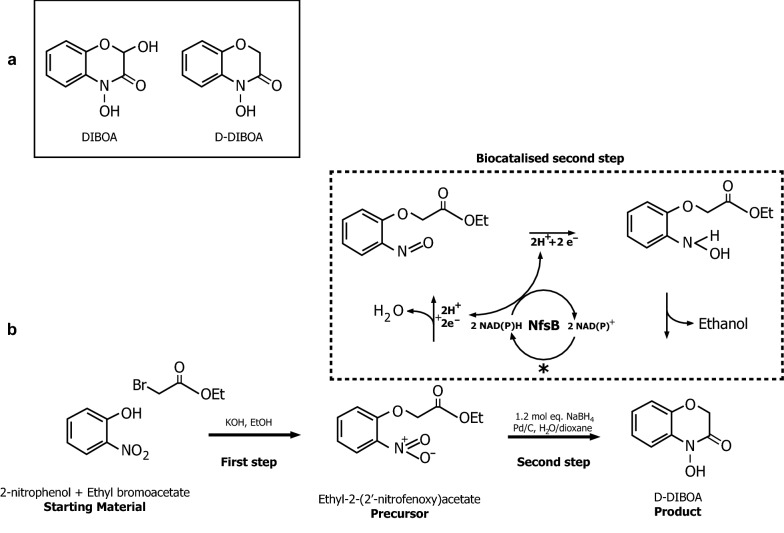
**a** DIBOA and D-DIBOA chemical structures. **b** D-DIBOA synthesis. The chemical synthesis of D-DIBOA was simplified into two steps; the first step is a nucleophilic substitution to introduce an ethyl bromoacetate chain using 2-nitrophenol as the starting material. The product of this reaction is ethyl 2-(2′-nitrophenoxy)acetate (precursor). The second step can be carried out in two ways; the chemical synthesis, which involves a heterogeneous catalysis with a Pd/C catalyst or a reaction catalysed by NfsB enzyme (dashed box), in which two NAD(P)H molecules are oxidized. The regeneration of these cofactors (*) is a limiting factor of the process

Benzohydroxamic acids is a group of these natural allelochemicals present in common agricultural crops such as wheat, rye and maize [9]. These compounds are well known for their interesting biological properties as herbicides, fungicides and insecticides [10, 11]. Among them, 2,4-dihydroxy-(2H)-1,4-benzoxazin-3-(4H)one (DIBOA) (Fig. 1a), a compound isolated from plant species belonging to the *Poaceae* family, has been proven to be a successful natural herbicide model [12], showing high biodegradability in soils and therefore a low environmental impact [10].

However, the main drawback for the use of DIBOA and other allelopathic compounds in agriculture is their very low natural production [13]. An alternative is the chemical synthesis, a process that reaches relatively high yields [6]. In the case of DIBOA, the synthesis of the biologically active analogous compound D-DIBOA (2-deoxy-DIBOA) has been achieved (Fig. 1a). The choice of D-DIBOA as a model for phytotoxic benzoxazinones resulted in significant improvements in phytotoxicity and selectivity on the species assayed (*Triticum aestivum* L. (wheat) and *Allium cepa * L. (onion), *Lycopersicon esculentum* Will. (tomato), *Lepidium sativum* L. (cress) and *Lactuca sativa* L. (lettuce). The N–OH moiety is a common feature in these compounds and probably constitutes the base of their biological activity. 2-Deoxy benzoxazinones derivatives display a wide range of activities, which makes these compounds one of the most interesting herbicide models [12]. The stability of benzoxazinones and their derivatives has been extensively investigated, especially for natural benzoxazinones and benzoxazolinones [10]. Changes in the aromatic substitution pattern markedly modify the stability of benzoxazinones and their derivatives [8, 12]. The half-life for D-DIBOA is slightly longer than that found for DIBOA and the chemicals belonging to its degradation series. In this sense, the first chemical of the D-DIBOA degradation series was also determined to be the lactam D-HBOA, which is slightly less phytotoxic than D-DIBOA [10].

The synthesis of D-DIBOA has been simplified into only two steps (Fig. 1b). The first one is carried out at relatively mild conditions using non-expensive starting materials (2-nitrophenol and ethyl bromoacetate) and reaches around 99% molar yield of ethyl-2-(2′-nitrophenoxy)acetate (which is the precursor of D-DIBOA and henceforth referred to as precursor). However, the second step, which involves the reduction of a nitro group followed by a cyclization, is an exothermic reaction with hydrogen release that requires NaBH_4_, dioxane and an expensive catalyst (Pd/C). Furthermore, the reaction yield (70%) is lower than that of the first reaction [6]. This methodology is therefore too expensive and difficult to be applied at higher scale.

This chemical reaction represents an example of the challenges that green-chemistry has been designed to address. Green-chemistry is aimed to develop processes in which desired products are maximized and undesired products are minimized, using environmentally friendly solvents in the procedures. In the past decade, many large chemical companies, have begun to use green-chemistry practices for the development and manufacturing of different products [14]. Biocatalysis based on enzymes is one of the most interesting strategies applied in green-chemistry because enzymes are very selective and lead to extremely high reaction rates at mild conditions, overcoming abiotic catalysts. This strategy has provided powerful tools in the synthetic chemist’s toolbox [15, 16].

The use of microorganisms as cell factories can be an environmentally friendly alternative for the synthesis of plant natural products such as DIBOA. Nevertheless, it is difficult to reproduce biosynthetic pathways of the natural products since the catalytic activity of heterologous plant enzymes is normally low. The fermentation costs, in most of the cases, are therefore too high for industrial-scale production due to the poor yields obtained from the use of these metabolic engineered microorganisms [17].

We propose in this work an alternative to the design of a multiple enzyme pathway for the synthesis of DIBOA. To this aim, the role of the microorganism is simplified by using it as a whole-cell biocatalyst for the synthesis of the synthetic analogue D-DIBOA. Thus, instead of the reconstruction of the whole DIBOA synthetic pathway, it is possible to replace the second step of D-DIBOA chemical synthesis by a biotransformation process carried out by an *E. coli* strain overexpressing the homologous nitroreductase enzyme NfsB. In a previous work we described how an *E. coli* strain overexpressing this enzyme was able to produce D-DIBOA from the precursor molecule at lower yield (60%) than the chemical synthesis using a non-defined culture medium [18]. This strain was also used as a whole-cell-biocatalyst for the synthesis of two chlorinates derivatives of D-DIBOA with similar molar yields [19]. Bacterial nitroreductases, such as NfsB, are flavoenzymes that catalyse the NAD(P)H-dependent reduction of the nitro groups on nitroaromatic and nitroheterocyclic compounds, which are toxic and mutagenic for living organisms, transforming them in less toxic molecules that are then exported out of the cell [20].

In this work, we have carried out a screening for improving D-DIBOA biotransformation yield by overexpressing the NfsB enzyme in *E. coli* single mutants, in which the knockout genes were related to metabolic pathways that use resources such as carbon, cofactor NAD(P)H, ATP, H^+^, electrons or energy consumption. We assumed that an increased availability of these resources should improve the biotransformation. This strategy allowed us to construct an optimized double mutant strain by genetic modification that proved to be a suitable background for NfsB overexpression and D-DIBOA synthesis. To achieve higher yields and concentrations of D-DIBOA, several loads of precursors were added to the culture medium. In addition, the culture medium was optimized to facilitate subsequent downstream purification procedures. All these improvements make the whole process more feasible to be scalable at industrial level.

## Materials and methods

### Bacterial strains as genetic backgrounds, plasmids and primers

*Escherichia coli* BW25113 was used as the wild type reference strain because this was the parental strain of the 34-isogenic single knockout strains used in this work (Table 1). These strains were originated in the Keio Collection from NAIST (Kyoto, Japan) [21] and purchased from the CGSC (Coli Genetic Stock Center), (Yale University, USA). The knockout genes were checked by PCR using the primers described in Table 2. The pBAD-NfsB inducible vector, previously cloned in our laboratory [18], was used to transform the wild type and the mutant strains used in the biotransformation assays.

**Table 1:** Knockout mutant genetic backgrounds assayed for D-DIBOA production

MultiFun Ontology: metabolism, child classes	Superpathways	Knock-out genes	ID (Keio collection)	Protein name	Pathways/reactions/molecular function	Consumed cofactor
Biosynthesis of Macromolecules (cellular constituents)^a^	Colanic acid (M antigen)	*rcsA*	JW1935-1	Rcsa	RcsA + RcsB → RcsAB	
					Positive DNA-binding transcriptional regulator of capsular polysaccharide synthesis, activates its own expression	
		*wzc*	JW2045-1	Protein-tyrosine kinase Wzc	ATP + a [protein]-l-tyrosine → ADP + a [protein]-l-tyrosine phosphate + H^+^	ATP
	Cytoplasmic poysaccharides	*dgsA (=mlc)*	JW1586-1	Mlc DNA-binding transcriptional repressor	Controls the expression of a number of genes encoding enzymes of the phosphotransferase (PTS) and phosphoenolpyruvate (PEP) systems. Regulates genes involved in the uptake of glucose	
		*glgA*	JW3392-1	Glycogen synthase	Glycogen biosynthesis I (from ADP-d-Glucose)	
	Enterobacterial common antigen (surface glycolipid)	*wzxE*	JW3766-1	Lipid III flippase	Enterobacterial common antigen)-undecaprenyl diphosphate[cytosol] → (enterobacterial common antigen)-undecaprenyl diphosphate[periplasm]	
	Flagellum	*flgA*	JW1059-1	Flagellar biosynthesis protein FlgA	Assembly of basal-body periplasmic P ring	ATP
		*flhA*	JW1868-1	Flagellar biosynthesis protein FlhA	Flagellar export apparatus	
		*fliQ*	JW1933-1	Flagellar biosynthesis protein FliQ		
	Transcriptional level, repressor^b^	*lrhA*	JW2284-6	LrhA DNA-binding transcriptional dual regulator A	LysR homologue A, regulates the transcription of genes involved in the synthesis of type 1 fimbriae. Indirectly, this protein also regulates the transcription of several genes involved in motility, chemotaxis, and flagellum synthesis by directly controlling the expression of the master regulator FlhDC	
	Large molecule carriers	*ccmA*	JW5366-1	Heme trafficking system ATP-binding protein	Ferroheme b[cytosol] + ATP + H_2_O → ferroheme b[periplasm] + ADP + phosphate + H^+^	ATP
		*citD*	JW0609-1	Citrate lyase, acyl carrier gamma; subunit	Citrate → acetate + oxaloacetate citrate + an acetyl-holo [citrate lyase acyl-carrier protein] + H ^+^ → a citryl-holo [citrate lyase acyl-carrier protein] + acetate a citryl-holo [citrate lyase acyl-carrier protein] → oxaloacetate + an acetyl-holo [citrate lyase acyl-carrier protein] + H^+^	
	Large molecule carriers, Thioredoxin, glutaredoxin	*grxA*	JW0833-1	Glutaredoxin 1, redox coenzyme for ribonucleotide reductase	2 glutathione + an oxidized glutaredoxin → glutathione disulfide + a reduced glutaredoxin an oxidized glutaredoxin + 2 e^-^ = a reduced glutaredoxin S-sulfo-l-cysteine + a reduced glutaredoxin → l-cysteine + sulfite + an oxidized glutaredoxin + H^+^ + 2 more	e^−^
	Lipopolysaccharide	*lapA (*= *yciS)*	JW1271-1	Lipopolysaccharide assembly protein LapA	Heat shock protein involved in the assembly of lipopolysaccharides	
	Lipopolysaccharide, core region	*kdsD*	JW3164-1	d-arabinose 5-phosphate isomerase	Aldehydo-d-arabinose 5-phosphate ↔ d-ribulose 5-phosphate	
		*lpcA*	JW0212-1	d-sedoheptulose 7-phosphate isomerase	d-sedoheptulose 7-phosphate → d-glycero-d-manno-heptose 7-phosphate	
	Lipopolysaccharide, Lipid A	*arnA*	JW2249-1	Fused UDP-L-Ara4 N formyltransferase and UDP-GlcA dehydrogenase	Polymyxin resistance	
	Lipopolysaccharide, O antigen	*rfbX*	JW2022-2	O antigen flippase	(O16 antigen)-undecaprenyl diphosphate_[cytosol]_ → (O16 antigen)-undecaprenyl diphosphate_[periplasm]_	
		*wbbI*	JW2019-1	β-1,6-galactofuranosyltransferase	Octyl α-d-glucopyranoside + UDP-α-d-galactofuranose → octyl β-1,6-d-galactofuranosyl-α-d-glucopyranoside + UDP + H^+^	
	Lipoprotein	*ybaY*	JW0443-1	Predicted outer membrane lipoprotein	Outer membrane component	
	Phospholipid	*cdsA*	GN80	CDP-diglyceride synthetase	CTP + a 1,2-diacyl-sn-glycerol 3-phosphate + H^+^ → a CDP-diacylglycerol + diphosphate CTP + a 2,3,4-saturated 1,2-diacyl-sn-glycerol 3-phosphate + H^+^ → a CDP-2,3,4-saturated-diacylglycerol + diphosphate	CTP/H^+^
		*clsA*	JW1241-5	Cardiolipin synthase 1	2 an L-1-phosphatidyl-sn-glycerol → a cardiolipin + glycerol	
		*pgpA*	JW0408-4	Phosphatidylglycerophosphatase A	1-(3-sn-phosphatidyl)-sn-glycerol 3-phosphate + H_2_O → an L-1-phosphatidyl-sn-glycerol + phosphate	
Carbon utilization	Fatty acids	*fadR*	JW1176-1	DNA-binding transcriptional dual regulator FadR	FadR + an acyl-CoA ↔ FadR-acyl-CoA	NADPH
	Carbon compounds	*pfk I*	JW3887-1	6-phosphofructokinase I	d-sedoheptulose 7-phosphate + ATP → d-sedoheptulose-1,7-bisphosphate + ADP + H^+^	NADH/ATP
		*pfk II*	JW5280-1	6-phosphofructokinase II	β-d-fructofuranose 6-phosphate + ATP → ADP + β-d-fructose 1,6-bisphosphate + H^+^	NADH/ATP
Central intermediary metabolism	Sugar nucleotide biosynthesis, conversions	*sthA (*= *udhA)*	JW5551-1	Pyridine nucleotide transhydrogenase, soluble	NAD^+^ + NADPH → NADH + NADP^+^	NADPH
Energy metabolism, carbon	Anaerobic respiration//electron donors	*nuoA*	JW2283-1	NADH:quinone oxidoreductase subunit A	NADH + ubiquinone _[inner membrane]_ + 5 H^+^ ↔ NAD^+^ + ubiquinol_[inner membrane]_ + 4 H^+^_[periplasm]_	NADH/H^+^
		*nuoB*	JW5875-2	NADH:ubiquinone oxidoreductase, chain B		
		*nuoE*	JW2280-1	NADH:ubiquinone oxidoreductase, chain E		
		*nuoF*	JW2279-3	NADH:ubiquinone oxidoreductase, chain F		
		*nuoG*	JW2278-1	NADH:ubiquinone oxidoreductase, chain G		
		*nuoN*	JW2271-1	NADH:ubiquinone oxidoreductase, membrane subunit N		
	Glycolysis	*pgi*	JW3985-1	Phosphoglucose isomerase	d-glucopyranose 6-phosphate ↔ β-d-fructofuranose 6-phosphate	
Energy production/transport	Transport → electron acceptors	*narG*	JW1215-1	Nitrate reductase A, alpha; subunit	Nitrate + 2H^+^ + menaquinol – > Nitrite + menaquinone + H_2_O	H^+^/e^−^

^a^Selected genes from a random search of biosynthesis of macromolecules as shown in Additional file 2. The other mutant strains have been selected from Table S1B

^b^Transcriptional protein repressor gene (*lrhA*) involved in flagellum, motility and chemotaxis related to flagellum protein not included in Metabolism class

**Table 2:** Primers used in this study for the PCR check and mutagenesis

Name	Description/Sequence (5´-3´)	Sources
*Primers*		
Kt-r^a^	CGGCCACAGTCGATGAATCC	Datsenko and Wanner [22]
rcsA-f	GTGACCCATGTTGTTCCGTTTAG	This study
wzc-f	CGCCATATCGAACGCTTATG	This study
dgsA-f	CCGTCATCACTCAGGAGGTG	This study
glgA-f	CCGGAACTGGATATGTACGATC	This study
wzxE-f	GCAGAACGCGCATATGTTCTAC	This study
flgA-f	ATTGCGGACAGGTACAATTCAC	This study
flhA-f	TGTATCGACATGCGGAGATTG	This study
fliQ-f	GTGATAGCCAGCGTGTTGATG	This study
lrhA-f	TGCACGAGAGTGGAACAAGG	This study
ccmA-f	CTGACGATGGCACAGAATGAG	This study
citD-f	CCAGGAGATGCCGATATCCGC	This study
grxA-f	GCTTCCCTCTGCAAAGTGAGCC	This study
lapA-f	GCGAACGTTAACCATTGCTATC	This study
lapA-r	AACGGAACAGGTTTCCGAGCG	This study
kdsD-f	CGTGACTACAGCGTGATGTTG	This study
lpcA-f	AGCACCTGCCCGTACTTCTCGC	This study
arnA-f	CTGACCTTCGGACCACAATG	This study
rfbX-f	TGGTTCTGTCTGATATCGCTG	This study
wbbI-f	ACAGGTGGAGTCTCTATGTCG	This study
ybaY-f	GGCAGAAATGCGTGATGTGTGC	This study
cdsA-f	GATGTTCTCTGGCCCGATTTC	This study
clsA-f	TCCGTTCTACTCCGCTTCATG	This study
pgpA-f	TCACTGTGCCGGAACTGAACC	This study
fadR-f	GCAGGAGTGAGGCAAGTCTTG	This study
pfk I-f	TGGTTCAGGCACATATGGTG	This study
pfk II-f	CATAACGATGGCAGGAACTGTC	This study
sthA-f	CCCATCACGATGTCTGAATCC	This study
nuoA-f	TATCCTGGAGTCGTCAAGGATC	This study
nuoB-f	TCGCATGGTCAACCTCTATCC	This study
nuoE-f	CAGAGTCTGCGCATTCTTGAG	This study
nuoF-f	GCGTGGTCTGTCATATCAACG	This study
nuoG-f	GTGCAGTGGAGCCGTTACAG	This study
nuoN-f	GGCAGCTTCCAGGTTGTACC	This study
pgi-f	CCTGTAGCCGATGATGAACG	This study
narG-f	CCACGCTGTTTCAGAGCGTTAC	This study
H1P4-fliQ	TGCTGGTCGGTTCGCTGGCGCAGAGCTTTTACAGCTAGAGAGGCAAAATGATTCCGGGGATCCGTCGACC	This study
H2P1-fliQ	GTTCGCTTGTCACCTGCAACATAGTACGGCTACCCGATGATATACGGCAGTGTAGGCTGGAGCTGCTTCG	This study
*Plasmids*		
pBAD-NfsB	pBAD vector with Amp^R^ harbouring the nitroreductase NfsB	Valle [18]
pCP20	Amp^R^ and Cm^R^ plasmid; thermal induction of FLP synthesis	Datsenko and Wanner [22]
pKD46	Amp^R^ plasmid contains red system for homologous recombination	Datsenko and Wanner [22]
pKD13	Kan^R^ plasmid; was used for generating the cassette with kanamycine resistance gene	Datsenko and Wanner [22]
*Constructed strain*		
*lapAfliQ*::kan	Kan^R^ mutant	This study

Ap^R^ , Kan^R^and Cm^R^ indicate ampicillin, kanamycin and chloramphenicol resistance

^a^All mutant strains were checked by the kt-r reverse primer of internal kanamycin resistance cassette [22]

### Construction of the *E. coli* ∆*lapA*∆*fliQ* double mutant strain

The construction of the **∆***lapA***∆***fliQ* double mutant was carried out following the homologous recombination method described by Datsenko and Wanner [22] using the *lapA*::kan single mutant as background strain and the primers 5′ (H1P4) and 3′ (H2P1) with the homologous sequences (H1 or H2) of the upstream and downstream *fliQ* gene flanking with P4 or P1 priming sequences of pKD13 vector (Table 2).

### Culture medium and chemicals

All the strains used in this work were grown in Luria–Bertani (LB) medium, LB agar plates and M9 minimal medium, containing in g/L: 0.24 MgSO_4_, 0.01 CaCl_2_, 11.178 Na_2_HPO_4_, 3.00 KH_2_PO_4_, 0.50 NaCl, 1.00 NH_4_Cl, 4.00 glucose and with or without 1.00 thiamine. Chemicals for culture media were purchased from Panreac. The media for the mutant strains were supplemented with 50 µg/mL kanamycin and the strains transformed with the pBAD-NfsB were supplemented with 100 µg/mL ampicillin. NfsB overexpression in the pBAD transformed strains was induced by adding 0.02% (w/v) l-arabinose. Antibiotics and l-arabinose were purchased from Sigma-Aldrich. Chemical standards of precursor and D-DIBOA were kindly provided by Allelopathy Group (Organic Chemistry Department, University of Cadiz) [6].

### Biotransformation assays

All the strains assayed in this work were streaked from a − 80 °C glycerol stock on LB agar plates and incubated overnight at 37 °C. A single colony was inoculated in 5 mL of LB medium and cultivated at 37 °C and 200 rpm in an orbital shaker. After 8 h, the cells were centrifuged at 3000*×g* for 10 min and the pellet was resuspended in 100 mL of LB or M9 medium containing 0.02% (w/v) l-arabinose to induce the expression of the *nfsB* gene and incubated overnight in the same conditions. 10 mL of this pre-culture were then centrifuged at 3000*×g* for 10 min and resuspended in 100 mL of fresh LB or M9 medium supplemented with 0.02% (w/v) l-arabinose and grown in 250 mL Erlenmeyer flask at 30 °C or 37 °C. The biotransformation assay was initiated by adding 1 mL of precursor stock solution (50 mg/mL in methanol) when OD_600_ = 0.6 (the initial precursor concentration at time 0 was therefore 0.5 mg/mL [2.22 mM]).

### Scanning electronic microscopy (SEM)

Bacteria at mid-exponential growth phase were diluted 1:2 and placed on polylysine cover slides and fixed with 2.5% glutaraldehyde in 0.1 M Na-cacodylate buffer (pH 7.2) for 1 h and washed twice for 10 min with the same buffer. After fixation, the samples were dehydrated in 70, 80, 90 and 100% ethanol solutions for 30 min and dried under critical point conditions in a Balzers Critical Point Dryer operated with liquid CO_2_. Gold particles were sputtered on the samples to avoid charging in the microscope. The images were taken at the Electron Microscopy Division of the Central Services of Scientific and Technological Research (SC-ICYT) from the University of Cadiz with a Nova NanoSEM 450 (FEI, Thermo Fisher), operating at voltage ranging from 5 to 10 kV.

### Analytical techniques, calculation of parameters and statistical analysis

Cell growth was estimated by measuring OD_600_ (U-2001 spectrophotometer HITACHI Instruments Inc. Tokyo). Biomass was estimated by the ratio, 1 OD_600_ = 0.33 g of cell dry weight (CDW)/L, according to standard procedure [23]. Additionally, for the toxicity test a Multiskan FC^®^ microplate reader with incubator (Thermo Scientific) was used to analyze the growth of different mutant strains. For quantitative analysis of precursor and D-DIBOA, 1 mL samples were withdrawn from the cultures and filtered through 0.22 µm nylon filters (VWR International) before analysis in a reverse-phase high performance liquid chromatograph (HPLC) (LaChrom Elite VWR-Hitachi) equipped with Phenomenex Gemini C18 4.6 × 250 mm column (Torrance, CA, USA) by using the method described in Valle et al. [18].

Biotransformation yield (BY) was calculated from the concentration of D-DIBOA at the biotransformation time assayed and the initial (i) precursor concentration:

$$BY = \frac{mol\,D - DIBOA}{{mol\,precursor_{i} }} \times 100$$

while specific productivity (SP) was defined as:

$$SP = \frac{mol\,D - DIBOA}{gCDW \times h}$$

Normalization of D-DIBOA concentration and SP parameters (*X*) for each replicate (Rn) was calculated as follows:

$$Xnorm_{Rn} = \frac{{Xmut_{Rn} - Xwt_{Rn} }}{{Xwt_{Rn} }}$$

Average and standard deviation were calculated using at least 3 replicates. Statgraphics Centurion software (Version XVII) was used to determine statistically significant differences between the group values by using *t*-Student’s test.

## Results and discussion

### D-DIBOA biotransformation in defined M9 medium

**Fig. 2:**
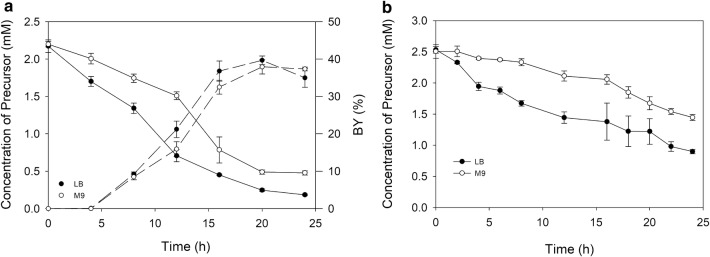
Study of biotransformation in two culture media. **a** Kinetic of the precursor biotransformation to D-DIBOA by the BW25113/pBAD-NfsB strain in LB and M9 medium. BY (%) (solid lines) and the concentrations of precursor (dashed lines) are represented in biotransformation processes carried out in LB (full dot) and M9 (empty dot). **b** Abiotic test to evaluate degradability of precursor in LB and M9 media

One of the key elements in the industrial application of the microorganisms as microbial cell factories is the use of a culture medium that simplifies and facilitates the downstream processing and product purification. In a previous work, we described how the **∆***nfsB*/pBAD-NfsB *E. coli* strain was able to produce D-DIBOA in LB medium [18]. This is a complex medium containing multiple nitrogen and carbon organic compounds included in the yeast extract and peptone that caused precursor degradation (Additional file 1) and could hinder forthcoming purification of the target product. In a recent study we demonstrated that the biotechnological production of chlorinated D-DIBOA derivatives using the **∆***nfsB*/pBAD-NfsB strain was feasible by using the minimum defined culture medium M9 [19]. For these reasons, in this work the biotransformation process by BW25113/pBAD-NfsB *E. coli* strain in M9 medium was evaluated and the results were compared to those obtained in LB medium (Fig. 2a). In these assays precursor concentration and BY were evaluated at 0, 4, 8, 12, 16, 20 and 24 h. The results of this analysis showed that, although the BY in both media were similar (40% for LB and 38% for M9 at 20 h), the precursor concentrations were lower in LB than in M9 at all of the times assayed. This would indicate that the precursor is in fact more stable in M9 medium than in LB (0.48 and 0.18 mM precursor concentrations at 24 h respectively), although this *E. coli* strain is not capable of biotransforming the available precursor.

To further characterize precursor stability, abiotic tests with M9, LB and the different components of the LB medium (yeast extract, peptone and NaCl) were carried out (Additional file 1). These tests showed how a mixture of peptone and yeast extract strongly affects to precursor stability. Peptone seems to be the main responsible for precursor degradation because precursor stability is similar in LB without yeast extract than in LB complete medium. This degradation may be caused by the interaction with unidentified peptides contained in those protein extracts.

On the other hand, BY and D-DIBOA concentration drop in LB but not in M9 at 24 h (Fig. 2b), which indicates that D-DIBOA is also more stable in M9 medium. For these reasons, M9 culture medium was used for further optimization of the process.

### Screening of *E. coli* single mutants for suitable genetic backgrounds to improve D-DIBOA production

The most important factor to improve D-DIBOA production is to enhance the biotransformation yield of the biocatalyzed second step described in the Fig. 1b. In a previous work, we showed how the ∆*nfsB/*pBAD-NfsB *E. coli* strain was able to biotransform precursor to D-DIBOA with 60% molar yield [18], which is lower than that of the chemical synthesis (70%). We reasoned that a genetic background in which the availability of the NADH and NADPH or other cofactors were higher or the transport of precursor and D-DIBOA through the membrane were facilitated, could improve the activity of the NfsB enzyme. This strategy was previously used for other substrate of this enzyme [24]. For this purpose, a genetic screening for a more favourable mutant background for D-DIBOA production was carried out. This screening was based on a search for genes involved in anabolic pathways that use resources such as carbon, cofactors like NAD(P)H, ATP, H^+^, electrons or energy consumption carried out in the EcoCyc database [25]. The results of this search were collected in superpathways included in two categories: “Biosynthesis of Macromolecules: Cellular Constituents” (Additional file 2) and superpathways involved in “Carbon and Energy Metabolism” (Additional file 3). Each of these categories was then subdivided in metabolic subclasses. For instance, the superpathways of the category “Biosynthesis of Macromolecules (cellular constituents)” (267 genes) included the synthesis of colanic acid, cytoplasmic polysaccharides, enterobacterial common antigen or flagellum, lipopolysaccharide, etc. Seventy-nine of these genes were discarded because they are essential and knockout mutant strains are not viable. From the rest of the 188 genes, 22 single mutants were randomly selected from those related to functions involved in motility, cell wall synthesis or pathogenicity, which theoretically would not impair the biotransformation process but could improve the availability of resources for the synthesis of D-DIBOA. A second set of mutants was selected from superpathways involved in “Carbon and Energy Metabolism”. In this case the terms selected were “Carbon Utilization” (399 genes), “Central Intermediary Metabolism” (278 genes), “Energy Metabolism, Carbon” (203 genes) and “Energy Production/Transport” (96 genes). These terms were also divided in subclasses, such as fatty acids, and carbon compounds (carbon utilization), sugar nucleotide biosynthesis conversion (central intermediary), aerobic respiration or glycolysis (energy metabolism, carbon) and electron acceptors (energy production/transport), etc. The total number of genes in these categories was much higher (976), but the selection was more specific since only 12 mutant strains were chosen on the base of NADH, NADPH or H^+^ utilization: (1) fatty acids biosynthesis (*fadR*), an NADPH consuming process; (2) the NADH-quinone oxido-reductase complex I of the electron transport chain (*nuoABEFGN* genes); (3) the pyridine transhydrogenase (*sthA*) that plays the role of reoxidation of NADPH (NADPH +NAD^+^ → NADH + NADP^+^); (4) carbon compounds and glycolysis genes (*pfkA, pfkB* and *pg*i) that are related to NADPH regeneration with glucose by the Pentose Phosphate Pathway (PPP) as described by Siedler et al. [26]. In this work, the authors demonstrate that the interruption of glycolysis by deletion of the genes encoding phosphofructokinase (PfkI, II) and/or phosphoglucose isomerase (*pgi*) is adequate for NADH generation in whole-cell biotransformations. Finally (5), we have also selected the nitrate reductase *narG* mutant connected to the menaquinone/menaquinol transmembrane protein located in transport electron chain. This reaction could be connected indirectly to the NADH metabolism.

**Fig. 3:**
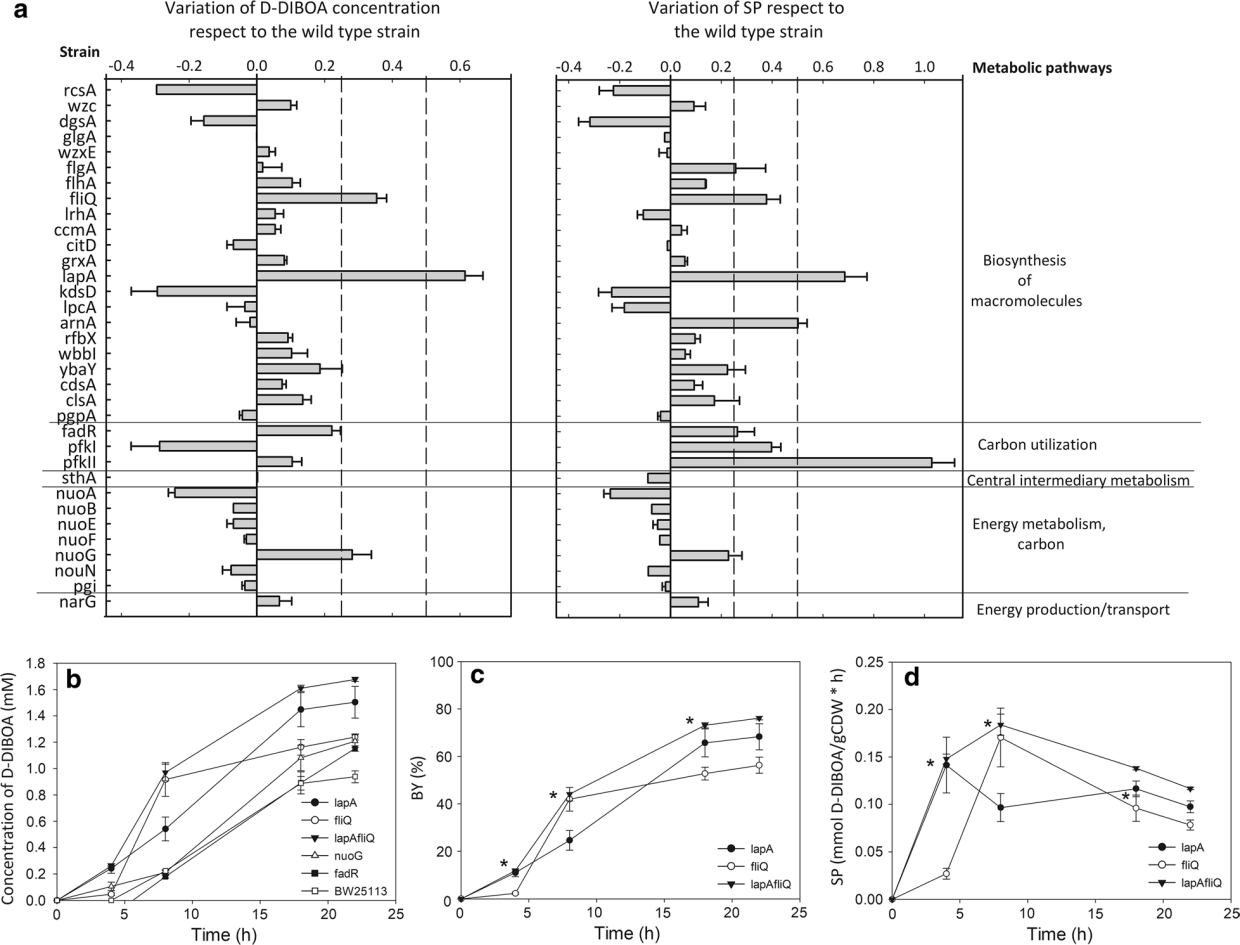
Screening of *E. coli* single mutant strains and optimization of the genetic background for D-DIBOA production. **a** Screening of *E. coli* single mutant strains transformed with the vector pBAD-NfsB for improved D-DIBOA synthesis. Bar charts show relativized values of D-DIBOA concentration (mM) and SP (mmol D-DIBOA/g_CDW_ x h) for single mutant strains respect to the wild type strain at 22 h. In no case the coefficient of variation were higher than 11%. In the left-hand column, the mutant strains assayed in this work are listed and in the right-hand column the metabolic pathways in which the mutated gene is involved are indicated. In order to facilitate the analysis of the screening, the mutant strains are listed in the same order as in Table 1. **b** Scatter plot of mean and SD (from at least 9 replicates) of D-DIBOA concentration in the reference, the single and double knockout strains. **c** BY and **d** SP for the single and double mutant strains under the screening conditions. Asterisks (*) show the pair of strains that have no statistically significant differences (*p* value > 0.05) for BY or SP. The parameters in **b**, **c** and **d** were evaluated at 0, 4, 8, 18 and 22 h time points

All of the selected mutant strains (Table 1), together with the wild type strain, used as control background, were transformed with the pBAD-NfsB inducible vector (Table 2). The mutant strains were then tested in biotransformation assays and D-DIBOA concentration and SP (Fig. 3a) were calculated and relativized respect to that obtained in the wild type strain at 22 h.

The screening strategy above described proved to be successful, since eight mutants in three of the tested metabolic categories showed values 25% higher for D-DIBOA concentration, SP or both parameters than those measured for the wild type strain. Remarkably, two of the mutant backgrounds from the category “Biosynthesis of macromolecules” showed the highest D-DIBOA concentrations in the culture medium. Thus, the *lapA* (lipopolysaccharide assembly protein A) mutant produced the highest concentration (60% higher than that of the wild type strain) as well as the second better SP (68%). In the case of the mutant *fliQ* (flagellar biosynthesis protein Q) the increments were 35% and 38% for BY and SP, respectively.

The third and fourth highest D-DIBOA producing strains were *nuoG* and *fadR* mutants, which are genes related to NADH (NuoG, a protein from the NADH-consuming complex I of the electron transport chain) and NADPH oxidation (FadR, a DNA regulator of the NADPH-consuming fatty acid synthesis). Both mutant strains showed concentration and SP higher than 20%. Several other strains showed high SP, but lower D-DIBOA concentrations (*pfkI*, *pfkII* and *arnA* mutants). This would indicate a favourable mutant background for NfsB function, although in these cases the combination of the mutation and NfsB overexpression seems to affect the bacterial growth. Consequently, the best D-DIBOA producer strains, (*lapA*, *fliQ*, *nuoG* and *fadR)* were selected for further analysis and optimization of the process.

### Kinetic of D-DIBOA production in the *lapA*, *fliQ*, *nuoG* and *fadR* mutant backgrounds

To further characterize the four best mutant backgrounds (*lapA, fliQ, nuoG* and *fadR*), D-DIBOA concentration was analysed at 0, 4, 8, 18 and 22 h in biotransformation experiments carried out by these strains (Fig. 3b). The analysis showed that, although all of them produced higher D-DIBOA concentration at 22 h than the BW25113/pBAD-NfsB reference strain, only in the case of **∆***lapA/*pBAD-NfsB this value was enhanced at all the time points tested. This strain also gave the highest final D-DIBOA concentration among the single mutant backgrounds (1.50 mM) and the highest BY (68%), increasing 60% respect to the reference strain values (Additional file 4). The other three single mutant strains showed a final production around 32% higher than that of the reference strain. However, in the case of the **∆***fliQ*/pBAD-NfsB strain the D-DIBOA concentration at 8 h was increased 311% respect to the wild type reference strain and almost doubled the D-DIBOA concentration obtained in the **∆***lapA*/pBAD-NfsB strain. This feature of *fliQ* mutant background was considered interesting since the **∆***nuoG* and **∆***fadR* backgrounds showed a similar behaviour to those of the wild type strain at intermediate times (Fig. 3b).

### The ∆*lapA*∆*fliQ/pBAD*-NfsB strain enhances D-DIBOA biotransformation yield up to 76%

One of the strategies commonly used to improve the yield of the biotransformation processes carried out by *E. coli* is to combine multiple mutations that individually enhance the production of the target product [27]. We hypothesized that, since the *lapA* and *fliQ* genes have unrelated functions with different kinetic behaviours in D-DIBOA production and both mutations did not affect cell growth, a double mutant of these genes could acquire the features of both single mutants, showing an increased production at 8 h and adding up the production of both strains.

To this end, the **∆***lapA***∆***fliQ* double mutant was constructed as described in Material and Method section and was transformed with the pBAD-NfsB vector. This strain was assayed at the same conditions described above for the single mutants. The analysis of the data obtained in these experiments revealed that indeed, D-DIBOA concentration, BY and SP were significantly improved respect to those obtained with the *lapA* and *fliQ* single mutant strains (Fig. 3b, c, d respectively). Furthermore, the double mutant strain showed complementary features of both single mutants, since all these parameters were enhanced at 8 h, at the same level as the *fliQ* mutant background and they were improved at the rest of the time points analysed like in the *lapA* mutant. In fact, the BY at 22 h in this new strain (76%) improved the single *lapA* (68%) and enhanced 16% the BY of the best previously reported strain (60%) [18] (Fig. 3c). In terms of SP, the double mutant also showed favourable features of both single mutants: *lapA* SP showed a maximum of productivity at 4 h while in the *fliQ* mutant the maximum SP, although higher, was reached at 8 h. Interestingly, in the case of the *lapAfliQ* double mutant the SP were virtually the same than in **∆***lapA* at 4 h and **∆***fliQ* at 8 h (Fig. 3d). Therefore, this double mutant seems to add up the positive features of both single mutants, showing not only a better D-DIBOA rate production and a higher final concentration, BY and SP than the single mutants, but also than the mutant strains previously reported by Valle et al. [18].

There are two critical elements that affects to a whole cell biocatalysis: on the one hand, internal factors such as enzyme concentration and cofactors availability and, on the other hand, the uptake of the substrate into the cytoplasm. In this work, mutants related to the biosynthesis of several macromolecule structures or related to theoretical increased NAD(P)H/NAD(P)^+^ ratio were screened. However, the two single mutants selected for their enhanced biotransformation capabilities were associated to the bacterial cell wall. This improved biocatalytic ability of the Δ*lapA*Δ*fliQ*/pBAD-NfsB strain may be because of a higher permeability to the precursor (substrate of the reaction).

**Fig. 4:**
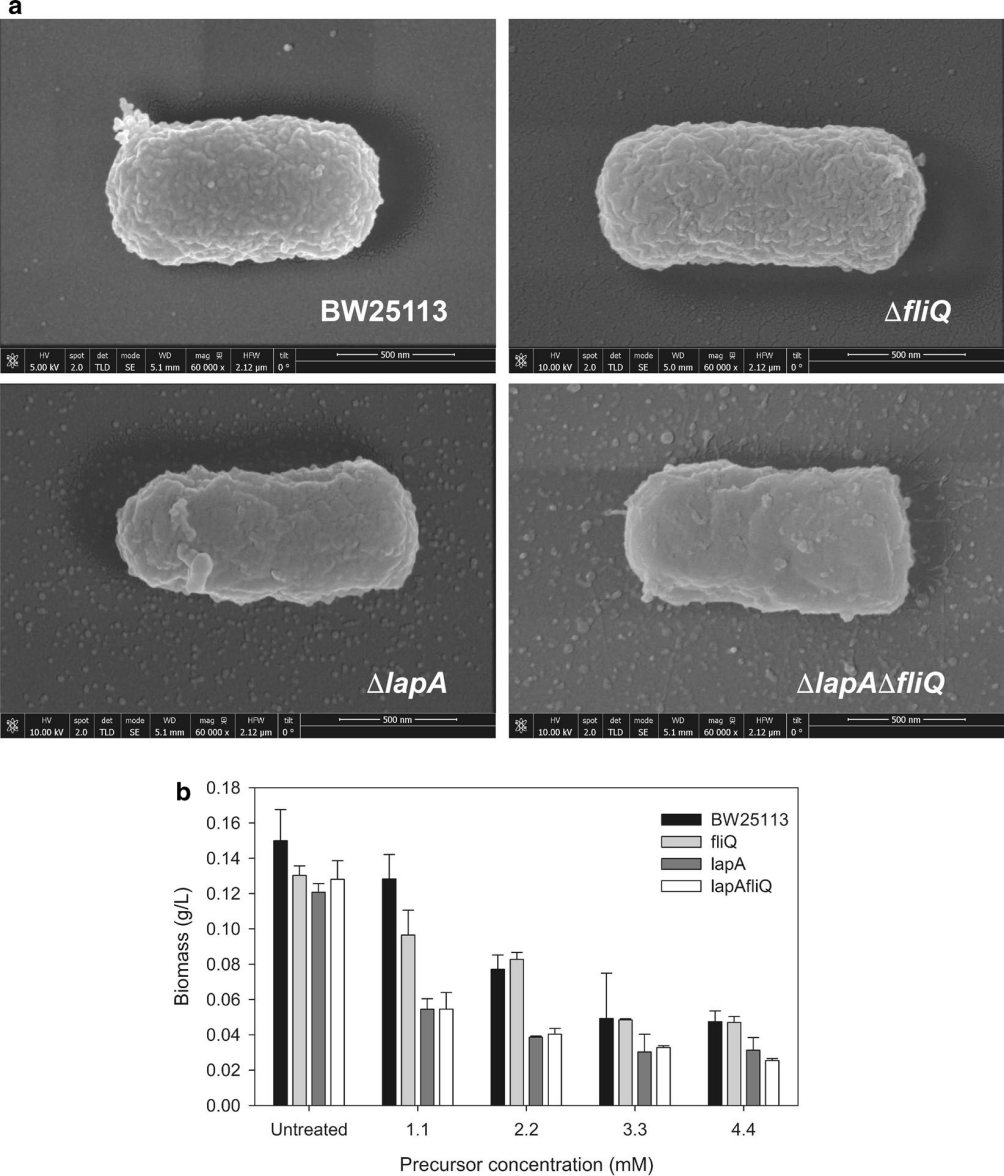
SEM micrographs of *E. coli* strains and tolerance test at 6 h in presence or absence of different concentrations of precursor. **a** SEM images for the wild type, the selected mutants and the double mutant strains overexpressing NfsB. **b** Growth tolerance test of the genetic backgrounds of these strains (without pBAD-NfsB) in presence of increasing concentrations of precursor

In this sense, the outer membrane (OM) of gram-negative bacteria, such as *E. coli*, acts as an effective permeability barrier against various toxic agents, including antibiotics. The diffusion of hydrophobic compounds through the enterobacterial OM is very restricted because of the lack of glycerophospholipid bilayers, the effective pathway for hydrophobic diffusion. The structural integrity of the OM is attributable to its unique lipopolysaccharide (LPS) constituent. Mutants that have a defective OM permeability barrier function are useful in various fields of basic and applied research and it has long been proposed that in biotechnology, bioconversion processes can greatly benefit from mutants that allow maximal diffusion of substrates [28]. This is probably the case of the *lapA* mutant. LapA (lipopolysaccharide assembly protein A) was previously described as a heat shock protein involved in the assembly of LPS in *E. coli*. Mass spectra of LPS from a Δ*lapA* mutant revealed accumulation of a few incomplete LPS precursors, although this strain can grow in M9 medium [29]. To investigate the effect of the elimination of this protein on the cell surface and on the permeability to the precursor, two different sets of experiments were carried out (Fig. 4). Firstly, scanning electronic microscopy on the wild type and the *lapA*, *fliQ* and the double mutant strains were performed. The aim of these experiments was to find out whether the LPS of the outer membrane were visibly affected. Indeed, we found that the normal rough surface and cylindrical tube shape with hemispherical caps of the *E. coli* wild type strain were altered in both the *lapA* and the double *lapA/fliQ* mutants (Fig. 4a). These changes were more evident in the double mutant strain in which most of the cells showed smooth surface and blunt ends. This would indicate an altered and more permeable OM to toxic compounds like the precursor used in this work. To test this hypothesis, the mutants and wild type background strains (without NfsB overexpression) were cultured with increased precursor concentrations and the bacterial growth was measured 6 h after precursor addition (Fig. 4b). Our results indicate that either the *lapA* or the double *lapA/fliQ* mutants showed lower cell growth even at the lowest precursor concentration assayed.

It is more difficult to explain the effect *fliQ* gene deletion on the biocatalysis. This protein is a component of the flagellar export apparatus that pass flagellar components from the cell membrane to the periplasm [30, 31]. It is worth to note that another mutant related to this export system, the mutant *flhA* also tested in the screening carried out in this work showed D-DIBOA concentration and SP 10% higher than those of the wild type strain. This advantageous phenotype was not observed in *flgA,* other component of the flagellum assembly not relate to the export machinery [32]. In this strain D-DIBOA concentration was similar to that of the wild type (Fig. 3a). There could therefore be a relation between this protein export system and a better ability of the NfsB enzyme to biotransform the precursor, although further research needs to be done to disclose this relation.

These results indicate that the strategy of screening of mutants for suitable genetic backgrounds in whole cell catalysis would be focused not only in cofactor requirements but also to LPS biosynthesis and transport to the OM. This would lead to improve diffusion of substrates to the interior of the cells. In this work we demonstrate that the flagellar assembly genes are also a potential source of favourable genetic backgrounds for biotransformation processes.

### The optimization of the culture conditions simplifies the process and increases the D-DIBOA biotransformation yield up to 90%

Since the engineered **∆***lap***∆***fliQ*/pBAD-NfsB strain proved to be suitable for D-DIBOA production, we optimized the culture conditions for this strain in order to improve yield and concentration and to reduce costs of the process by studying several operational variables. To this aim, the following parameters were studied at the same time points previously described and were optimized in the following hierarchical order:

#### Temperature

**Fig. 5:**
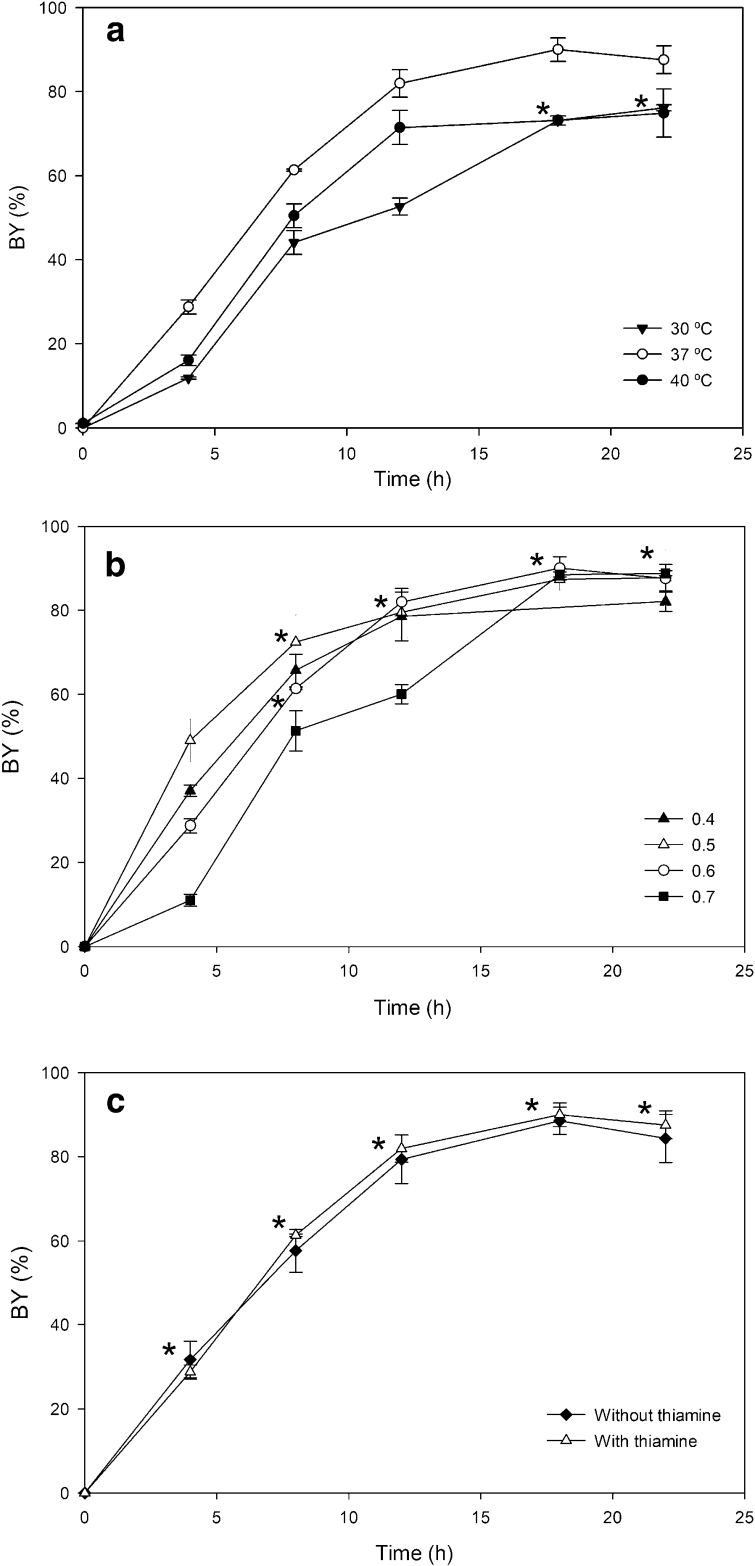
Optimization of three parameters for improving BY in the ∆*lap*∆*fliQ*/pBAD-NfsB strain. **a** Temperature. **b** Biomass at the time of addition of precursor to the culture medium (OD_600_) (biotransformation time point 0 h) **c** Thiamine supplementation. (*) indicates no statistically significant differences between experimental conditions at the times indicated

In previous works D-DIBOA biosynthesis was carried out at 30 °C to avoid precursor degradation, because its stability decreases with temperature in LB medium [18, 33]. Nevertheless, since the optimized strain is very efficient and the stability of precursor and D-DIBOA in the M9 culture medium was higher, we decided to study the synthesis of the target product at 37 °C and 40 °C. This analysis showed that the optimal temperature for the biotransformation was 37 °C, at which 90% BY at 18 h was obtained (Fig. 5a). In contrast, the BY obtained in cultures grown at 30 and 40 °C were lower (80% BY) and did not show statistically significant differences between them.

#### Inoculum biomass

Once the temperature of the process was optimized, we evaluated the effect of different biomass concentrations at the time in which the precursor was added to the culture medium (considered as starting time of the biotransformation) (Fig. 5b). We found that an increment in the OD, respect to that used in the screening (OD_600_ = 0.6), to 0.7 caused a decrease in the BY during the first 12 h. Nevertheless, a decrease in the initial OD to 0.5 produced an increment in the D-DIBOA production at the same time; although no significant differences at 18 h and 22 h were found. A further reduction in the OD to 0.4, showed lower BY than at 0.5 in the initial phase of the biotransformation and similar values at the end of the biotransformation assay. Therefore, an OD_600_ = 0.5 at 0 h was considered the optimum, since the kinetic behaviour of the biotransformation was improved and less biomass was needed.

#### Thiamine requirement

The M9 culture medium used in the screening and the others experiments described above was supplemented with thiamine. This is the most expensive component of the medium and it would increase the cost of the biological production of D-DIBOA at a higher scale. For this reason, the biotransformation in M9 with or without thiamine supplementation was tested. Our results showed that there were no statistically significant differences in BY neither in presence nor absence of thiamine in the culture medium (Fig. 5c). The use of this modified M9 medium would therefore reduce the costs and facilitate procedures for the scale up of the process.

### Successive precursor loads enhance D-DIBOA concentration in the culture medium up to 5 mM and increases the biotransformation yield up to 100%

**Fig. 6:**
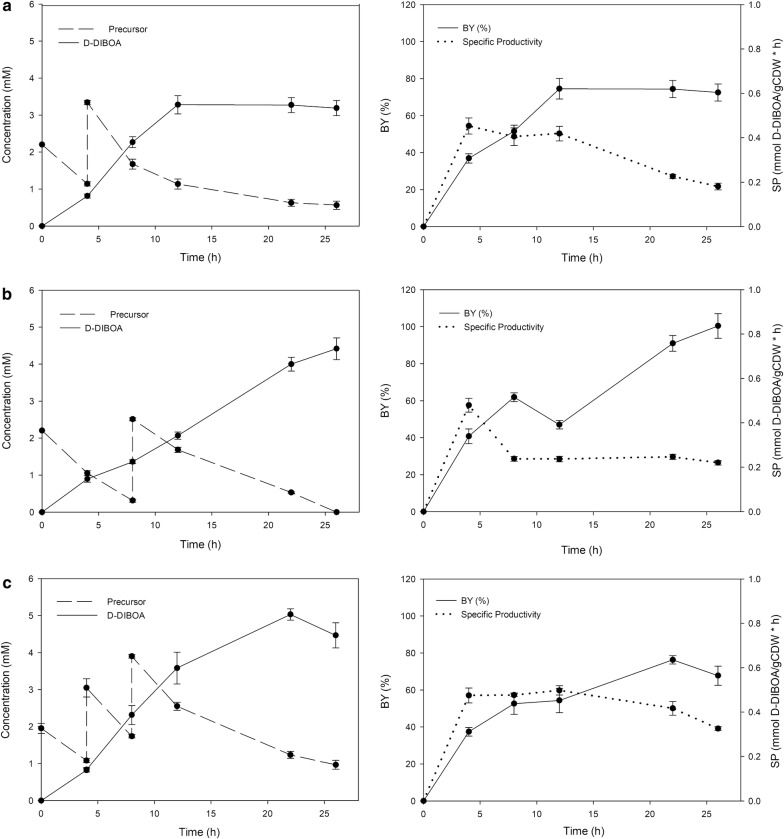
Biotransformation experiments with successive precursor loads. The culture media was fed with precursor doses in different addition times. Loads at **a** 0 and 4 h; **b** 0 and 8 h; **c** 0, 4 and 8 h. On the left, the concentration of precursor and D-DIBOA. On the right, the BY and SP

**Fig. 7:**
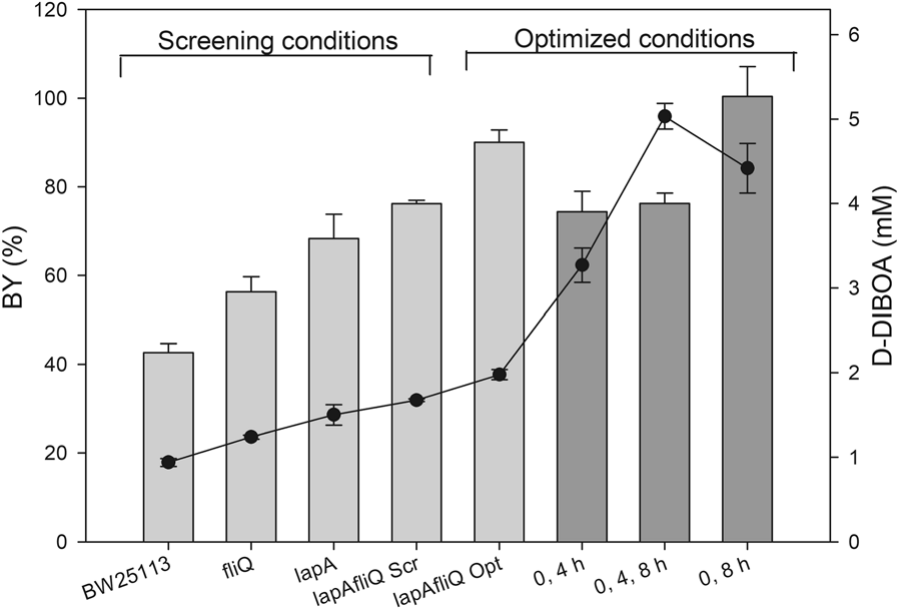
Summary of the main parameters of the biocatalysis carried out by the most relevant strains used in this work. BY (bars) and concentrations (line) reached in the biotransformation of precursor to D-DIBOA. All the strains indicated in the X-axis were transformed with the pBAD-NfsB vector, although for simplification, only the genetic mutant backgrounds of the strains are shown. Light grey indicates batch cultures for the knockout simple and double mutants and dark grey shows the cultures with successive precursor loads using the ∆*lap*∆*fliQ* double mutant

The industrial application of D-DIBOA biosynthesis requires not only maximum BY but also the highest possible concentration of the product in the culture medium. This could be achieved by increasing the initial amount of precursor added to the culture medium, however, the toxicity of the precursor is a limiting factor to this approach [33]. Nevertheless, the precursor is transformed into D-DIBOA overtime and we reasoned that several precursor loads at different times should avoid toxic precursor concentrations while would increase D-DIBOA concentration. For this reason, precursor reload assays were carried out by adding the same amount (0.5 mg/mL) at two time points selected on base of precursor concentration: when 50% (at 4 h) and 80% (at 8 h) of the initial precursor had been already consumed. Therefore, three different sets of experiments were carried out feeding the system at 0 h and then at 4 h or at 8 h or at both times. The analysis of these experiments (Figs. 6 and 7) shows how when the system was fed at 0 and 4 h (Fig. 6a), the production of D-DIBOA, was considerably higher than that of the batch system (3.27 mM versus 1.98 mM), although led to a considerable decrease of the BY (74%). However, remarkably, when precursor was fed at 0 and 8 h (Fig. 6b), all the precursor added to the culture medium was transformed into D-DIBOA at 26 h (BY = 100%) and the concentration (4.44 mM) was 300% higher respect to the data previously reported by Valle et al. [18] and more than 220% higher than that of the single fed assays described in this work. On the other hand, the experiment with three successive loads (Fig. 6c) produced the highest concentration of D-DIBOA described in a biotransformation synthesis so far (5.05 mM). This concentration is 379% respect to the highest concentration previously reported for the biological production of D-DIBOA (1.32 mM) [18]. Nevertheless, the biotransformation yields decreased to 76%. The growth curves for the three strategies have been included in the Additional file 5.

Therefore, a future scaling up of D-DIBOA bio-production would be based on systems with 2 loads: at 0 h (2.22 mM) and when precursor concentration decreases to 80%. In order to obtain a higher concentration of the target product, the system with three loads could be applied, but in this case, the precursor that remains unreacted in the biotransformation should be recovered in the D-DIBOA downstream process. With the aim of summarizing the improvements achieved in this work, the yields and concentrations obtained in all the optimization steps are shown in Fig. 7.

## Conclusions

In this work, we have engineered by genetic modification a novel *E. coli* strain that is capable of producing D-DIBOA with 100% yield from the chemical precursor. The use of a defined medium with only an organic molecule (glucose) in its composition will facilitate the product purification processes. An approach using successive precursor loads at lab scale has also increased the final D-DIBOA concentration in the culture medium around 300% respect to the biotransformation system previously reported. All of these improvements make the whole system feasible for the scaling up of the process.

## Additional files

**Additional file 1.** Analysis of precursor stability in different solutions and culture media. Precursor stability was measured by HPLC after 24 h of incubation in the same conditions for the biocatalysis described in the material and method section. Precursor concentration was measured in solutions containing peptone and yeast extract (Pept+YE), LB medium (LB), peptone and sodium chloride (Pept+NaCl) and M9 minimal medium supplemented with glucose (MM+Gluc).

**Additional file 2.** List of superpathways and genes related to biosynthesis of macromolecules: Cellular constituents (257 genes).

**Additional file 3.** List of the selected superpathways and genes involved in carbon and energy metabolism.

**Additional file 4.** Biotransformation yields in the biocatalysis carried out by ∆*lapA*, ∆*fliQ*, ∆*nuoG* and ∆*fadR* single mutant strains, ∆*lapA*∆*fliQ* double mutant strain and wild type strain at 4, 8, 18 and 24 h of experiment after precursor addition.

**Additional file 5.** Growth curves for: (a) Biotransformations with the *ΔlapA*, *ΔfliQ* and* ΔlapAΔfliQ* strains described in Fig. 3c and d. (b) Biotransformation with the *ΔlapAΔfliQ* double mutant in the multiple loads of precursor experiments described in Fig. 6.

## Data Availability

Not applicable.

## Abbreviations

BY: biotransformation yield
DIBOA: 2,4-dihydroxy-(2H)-1,4-benzoxazin-3(4H)-one
D-DIBOA: 4-hydroxy-(2H)-1,4-benzoxazin-3(4H)-one
HPLC: high performance liquid chromatography
NADH: nicotinamide adenine dinucleotide reduced
NADPH: nicotinamide adenine dinucleotide phosphate reduced
NfsB: NAD(P)H-dependent nitroreductase
SP: specific productivity

## References

[1] Owen MD, Zelaya IA (2005). Herbicide-resistant crops and weed resistance to herbicides. Pest Manag Sci.

[2] Kropff M, Walter H (2000). EWRS and the challenges for weed research at the start of a new millennium. Weed Res.

[3] Mortensen DA, Bastiaans L, Sattin M (2000). The role of ecology in the development of weed management systems: an outlook. Weed Res.

[4] Macías FA, Castellano D, Molinillo JM (2000). Search for a standard phytotoxic bioassay for allelochemicals. Selection of standard target species. J Agric Food Chem.

[5] Macías FA, Marín D, Oliveros-Bastidas A, Molinillo JMG (2009). Rediscovering the bioactivity and ecological role of 1,4-benzoxazinones. Nat Prod Rep.

[6] Macías FA, De Siqueira JM, Chinchilla N, Marín D, Varela RM, Molinillo JMG (2006). New herbicide models from benzoxazinones: aromatic ring functionalization effects. J Agric Food Chem.

[7] Rimando AM, Duke SO (2006). Natural products for pest management.

[8] Macías FA, Oliveros-Bastidas A, Marín D, Castellano D, Simonet AM, Molinillo JMG (2004). Degradation studies on benzoxazinoids. Soil degradation dynamics of 2,4-dihydroxy-7-methoxy-(2 H)-1,4-benzoxazin-3(4 H)-one (DIMBOA) and its degradation products, phytotoxic allelochemicals from gramineae. J Agric Food Chem.

[9] Fomsgaard IS, Mortensen AG, Carlsen SCK (2004). Microbial transformation products of benzoxazolinone and benzoxazinone allelochemicals—a review. Chemosphere.

[10] Chinchilla N, Marín D, Oliveros-Bastidas A, Molinillo JMG, Macías FA (2015). Soil biodegradation of a benzoxazinone analog proposed as a natural products-based herbicide. Plant Soil.

[11] Macías FA, Marín D, Oliveros-Bastidas A, Castellano D, Simonet AM, Molinillo JMG (2006). Structure–activity relationship (SAR) studies of benzoxazinones, their degradation products, and analogues. Phytotoxicity on problematic weeds *Avena fatua* L. and *Lolium rigidum* Gaud. J Agric Food Chem.

[12] Macías FA, Marín D, Oliveros-Bastidas A, Castellano D, Simonet AM, Molinillo JMG (2005). Structure–activity relationships (SAR) studies of benzoxazinones, their degradation products and analogues. Phytotoxicity on standard target species (STS). J Agric Food Chem.

[13] Macías FA, Marín D, Oliveros-Bastidas A, Chinchilla D, Simonet AM, Molinillo JMG (2006). Isolation and synthesis of allelochemicals from gramineae: benzoxazinones and related compounds. J Agric Food Chem.

[14] Tucker JL, Faul MM (2016). Drug companies must adopt green chemistry. Nature.

[15] Li C-J, Trost BM (2008). Green chemistry for chemical synthesis. Proc Natl Acad Sci.

[16] Hammer SC, Knight AM, Arnold FH (2017). Design and evolution of enzymes for non-natural chemistry. Curr Opin Green Sustain Chem.

[17] Tufvesson PR, Lima-Ramos J, Haque NA, Gernaey KV, Woodley JM (2013). Advances in the process development of biocatalytic processes. Org Process Res Dev.

[18] Valle A, Le Borgne S, Bolívar J, Cabrera G, Cantero D (2012). Study of the role played by NfsA, NfsB nitroreductase and NemA flavin reductase from *Escherichia coli* in the conversion of ethyl 2-(2`- nitrophenoxy)acetate to 4-hydroxy-(2H)-1,4-benzoxazin-3(4H)-one (D-DIBOA), a benzohydroxamic acid with interesting biological properties. Appl Microbiol Biotechnol.

[19] de la Calle ME, Cabrera G, Cantero D, Valle A, Bolivar J (2019). Overexpression of the nitroreductase NfsB in an *E. coli* strain as a whole-cell biocatalyst for the production of chlorinated analogues of the natural herbicide DIBOA. N Biotechnol.

[20] Roldán MD, Pérez-Reinado E, Castillo F, Moreno-Vivián C (2008). Reduction of polynitroaromatic compounds: the bacterial nitroreductases. FEMS Microbiol Rev.

[21] Baba T, Ara T, Hasegawa M, Takai Y, Okumura Y, Baba M (2006). Construction of E*scherichia coli* K-12 in-frame, single-gene knockout mutants: the Keio collection. Mol Syst Biol.

[22] Datsenko KA, Wanner BL (2000). One-step inactivation of chromosomal genes in *Escherichia coli* K-12 using PCR products. Proc Natl Acad Sci U S A.

[23] Greenberg AE, Clesceri L, Eaton A (1992). Standard methods for the examination of water and wastewater.

[24] Mercier C, Chalansonnet V, Orenga S, Gilbert C (2013). Characteristics of major *Escherichia coli* reductases involved in aerobic nitro and azo reduction. J Appl Microbiol.

[25] Keseler IM, Collado-Vides J, Gama-Castro S, Ingraham J, Paley S, Paulsen IT (2005). EcoCyc: a comprehensive database resource for *Escherichia coli*. Nucleic Acids Res.

[26] Siedler S, Bringer S, Bott M (2011). Increased NADPH availability in *Escherichia coli*: improvement of the product per glucose ratio in reductive whole-cell biotransformation. Appl Microbiol Biotechnol.

[27] Valle A, Cabrera G, Cantero D, Bolivar J (2015). Identification of enhanced hydrogen and ethanol *Escherichia coli* producer strains in a glycerol-based medium by screening in single-knock out mutant collections. Microb Cell Fact..

[28] Vaara M (1993). Antibiotic-supersusceptible mutants of *Escherichia coli* and S*almonella typhimurium*. Antimicrob Agents Chemother.

[29] Klein G, Kobylak N, Lindner B, Stupak A, Raina S (2014). Assembly of lipopolysaccharide in *Escherichia coli* requires the essential LapB heat shock protein. J Biol Chem.

[30] Berg HC (2003). The rotary motor of bacterial flagella. Annu Rev Biochem.

[31] Minamino T, Macnab RM (1999). Components of the Salmonella flagellar export apparatus and classification of export substrates. J Bacteriol.

[32] Kutsukake K, Nambu T (2000). The Salmonella FlgA protein, a putative periplasmic chaperone essential for flagellar P-ring formation. Microbiology.

[33] Valle A, Cabrera G, Molinillo JMG, Gómez JM, Macías FA, Cantero D (2011). Biotransformation of ethyl 2-(2`-nitrophenoxy)acetate to benzohydroxamic acid (D-DIBOA) by *Escherichia coli*. Process Biochem.

