# Supplementary material for: A genetically engineered Escherichia coli strain overexpressing the nitroreductase NfsB is capable of producing the herbicide D-DIBOA with 100% molar yield
Source: Microb Cell Fact. 2019 May 20;18:86. doi: 10.1186/s12934-019-1135-8 (PMC6526606; doi:10.1186/s12934-019-1135-8)

Additional file 1. Analysis of precursor stability in different solutions and culture media. Precursor stability was measured by HPLC after 24 h of incubation in the same conditions for the biocatalysis described in the material and method section. Precursor concentration was measured in solutions containing peptone and yeast extract (Pept+YE), LB medium (LB), peptone and sodium chloride (Pept+NaCl) and M9 minimal medium supplemented with glucose (MM+Gluc).

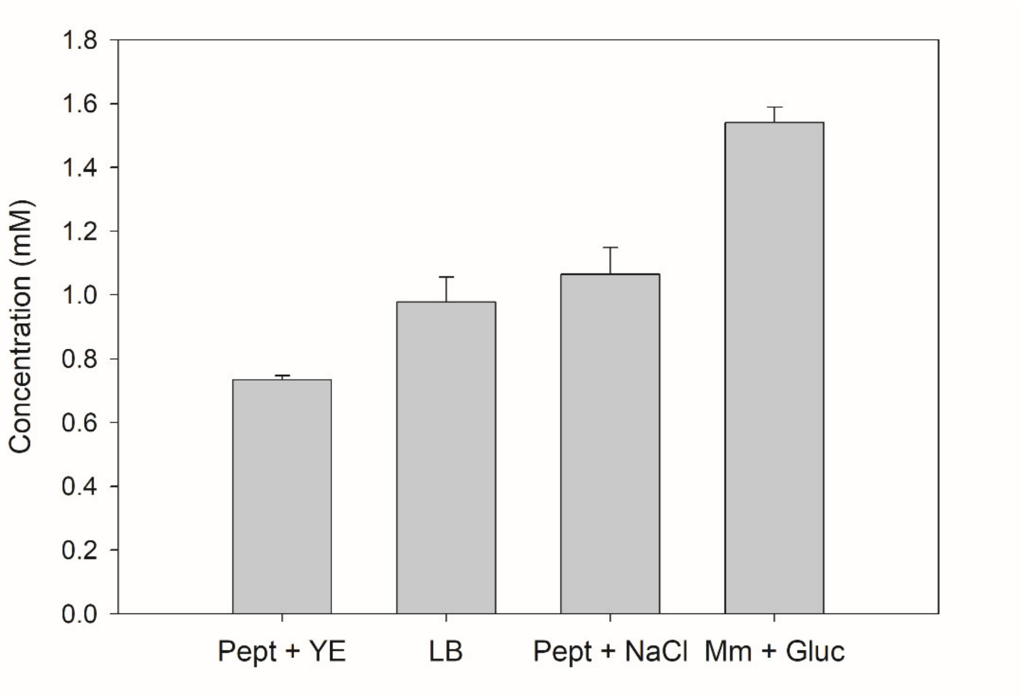

Supplement: Supplementary file 1 — Additional file 1. Analysis of precursor stability in different solutions and culture media. Precursor stability was measured by HPLC after 24 h of incubation in the same conditions for the biocatalysis described in the material and method section. Precursor concentration was measured in solutions containing peptone and yeast extract (Pept+YE), LB medium (LB), peptone and sodium chloride (Pept+NaCl) and M9 minimal medium supplemented with glucose (MM+Gluc). [file 12934_2019_1135_MOESM1_ESM.pdf]
