# Supplementary material for: A genetically engineered Escherichia coli strain overexpressing the nitroreductase NfsB is capable of producing the herbicide D-DIBOA with 100% molar yield
Source: Microb Cell Fact. 2019 May 20;18:86. doi: 10.1186/s12934-019-1135-8 (PMC6526606; doi:10.1186/s12934-019-1135-8)
Supplement: Supplementary file 2 — Additional file 2. List of superpathways and genes related to biosynthesis of macromolecules: Cellular constituents (257 genes). [file 12934_2019_1135_MOESM2_ESM.pdf]

Additional file 2. List of superpathways and genes related to biosynthesis of macromolecules:

Cellular constituents (257 genes)

Superpathway (number of genes)	Genes
Colanic acid (M antigen) (29)	cpsB (mannose-1-phosphate guanylyltransferase),<br>cpsG (phosphomannomutase),<br>fcl (GDP-fucose synthase),<br>galE (UDP-glucose 4-epimerase),<br>galK (galactokinase),<br>galT (galactose-1-phosphate uridylyltransferase),<br>galU (UTP—glucose-1-phosphate uridylyltransferase),<br>gmd (GDP-mannose 4,6-dehydratase),<br>gmm (GDP-mannose mannosyl hydrolase),<br>manA (mannose-6-phosphate isomerase),<br>rcsA (DNA-binding transcriptional activator RcsA),<br>rcsB (P^{asp56} RcsB),<br>rcsC (sensory histidine kinase RcsC - asp875 phosphorylated),<br>rcsF (outer membrane lipoprotein RcsF),<br>ugd (UDP-glucose 6-dehydrogenase),<br>wcaA (putative colanic acid biosynthesis glycosyl transferase),<br>wcaB (putative colanic acid biosynthesis acyl transferase),<br>wcaC (putative colanic acid biosynthesis glycosyl transferase),<br>wcaE (putative colanic acid biosynthesis glycosyl transferase),<br>wcaF (putative acyl transferase),<br>wcaI (putative colanic biosynthesis glycosyl transferase),<br>wcaJ (undecaprenyl-phosphate glucose phosphotransferase),<br>wcaK (putative colanic acid biosynthesis pyruvyl transferase),<br>wcaL (putative colanic biosynthesis glycosyl transferase),<br>wcaM (putative colanic acid biosynthesis protein WcaM),<br>wza (outer membrane polysaccharide export protein Wza),<br>wzb (protein-tyrosine phosphatase),<br>wzc (protein-tyrosine kinase Wzc),<br>ypdI (colanic acid synthesis putative lipoprotein YpdI)
Cytoplasmic polysaccharides (8)	csrA (carbon storage regulator),<br>glgA (glycogen synthase),<br>glgB (1,4-α-glucan branching enzyme),<br>glgC (glucose-1-phosphate adenylyltransferase),<br>glgP (glycogen phosphorylase),<br>glgS (surface composition regulator),<br>mlc (DNA-binding transcriptional repressor Mlc),<br>yaiP (putative glucosyltransferase)
Enterobacterial common antigen (surface glycolipid) (13)	glmM (phosphoglucosamine mutase),<br>glmU (fused N-acetylglucosamine-1-phosphate uridyltransferase and glucosamine-1-phosphate acetyltransferase),<br>rfe (UDP-*N*-acetylglucosamine—undecaprenyl-phosphate *N*-acetylglucosaminephosphotransferase),<br>rffG (dTDP-glucose 4,6-dehydratase 2),

	rffH (dTDP-glucose pyrophosphorylase 2),<br>rffM (UDP-N-acetyl-D-mannosaminuronic acid transferase),<br>rffT (4-acetamido-4,6-dideoxy-D-galactose transferase),<br>wecB (UDP-*N*-acetylglucosamine 2-epimerase),<br>wecC (UDP-*N*-acetyl-D-mannosamine dehydrogenase),<br>wecE (dTDP-4-dehydro-6-deoxy-D-glucose transaminase),<br>wzxE (lipid III flippase),<br>wzyE (enterobacterial common antigen polymerase),<br>wzzE (enterobacterial common antigen polysaccharide co-polymerase)
Fimbri, pili (5)	csgA (curlin, major subunit),<br>csgC (inhibitor of CsgA amyloid formation),<br>fimB (regulator for fimA),<br>fimE (regulator for fimA),<br>rfaH (transcription antiterminator RfaH)
Flagellum (32)	flgA (flagellar basal body P-ring formation protein FlgA),<br>flgB (flagellar basal-body rod protein FlgB),<br>flgC (flagellar basal-body rod protein FlgC),<br>flgD (flagellar biosynthesis, initiation of hook assembly),<br>flgE (flagellar hook protein FlgE),<br>flgF (flagellar basal-body rod protein FlgF),<br>flgG (flagellar basal-body rod protein FlgG),<br>flgH (flagellar L-ring protein),<br>flgI (flagellar P-ring protein),<br>flgJ (putative peptidoglycan hydrolase FlgJ),<br>flgK (flagellar hook-filament junction protein 1),<br>flgL (flagellar hook-filament junction protein 2),<br>flgN (flagellar biosynthesis protein FlgN),<br>flhA (flagellar biosynthesis protein FlhA),<br>flhC (DNA-binding transcriptional dual regulator FlhC),<br>flhD (DNA-binding transcriptional dual regulator FlhD),<br>fliC (flagellar filament structural protein),<br>fliD (flagellar filament capping protein),<br>fliE (flagellar basal-body protein FliE),<br>fliF (flagellar basal-body MS-ring and collar protein),<br>fliG (flagellar motor switch protein FliG),<br>fliH (flagellar biosynthesis protein FliH),<br>fliJ (flagellar biosynthesis protein FliJ),<br>fliK (flagellar hook-length control protein),<br>fliL (flagellar protein FliL),<br>fliM (flagellar motor switch protein FliM),<br>fliN (flagellar motor switch protein FliN),<br>fliO (flagellar biosynthesis protein FliO),<br>fliP (flagellar biosynthesis protein FliP),<br>fliQ (flagellar biosynthesis protein FliQ),<br>fliS (flagellar biosynthesis protein FliS),<br>fliT (flagellar biosynthesis protein FliT)
Glycoprotein (3)	csgB (curlin, minor subunit),<br>fimH (type 1 fimbriae D-mannose specific adhesin),<br>fliQ (flagellar biosynthesis protein FliQ)
K antigen (3)	lpxK (tetraacyldisaccharide 4'-kinase),<br>rcsA (DNA-binding transcriptional activator RcsA),<br>waaA (KDO transferase)
Large molecule carriers (37)	acyl carrier protein (3)<br>acpH (acyl carrier protein phosphodiesterase)<br>acpP (octanoyl-ACP),<br>citD (citrate lyase acyl carrier protein)<br>biotin carboxyl carrier protein (1)

	accB (biotin carboxyl carrier protein)<br>Cytochromes (26)<br>ccmA (heme trafficking system ATP-binding protein),<br>ccmB (heme trafficking system membrane protein CcmB),<br>ccmC (heme trafficking system membrane protein CcmC),<br>ccmD (heme trafficking system membrane protein CcmD),<br>ccmE (periplasmic heme chaperone),<br>ccmF (holocytochrome *c* synthetase membrane subunit CcmF),<br>ccmG (holocytochrome *c* synthetase - thiol:disulfide oxidoreductase CcmG),<br>ccmH (holocytochrome *c* synthetase - thiol:disulfide oxidoreductase CcmH),<br>cybB (cytochrome b561),<br>dsbD (protein disulfide oxidoreductase - $DsbD_{oxidized}$),<br>fdnI (formate dehydrogenase N subunit γ),<br>fdoI (formate dehydrogenase O subunit γ),<br>hyaC (hydrogenase 1 cytochrome *b* subunit),<br>hybB (hydrogenase 2 membrane subunit),<br>napB (periplasmic nitrate reductase cytochrome c_{550} protein),<br>napC (periplasmic nitrate reductase cytochrome c protein),<br>narI (nitrate reductase A subunit γ),<br>nrfA (cytochrome c_{552} nitrite reductase),<br>nrfB (periplasmic nitrite reductase penta-heme *c*-type cytochrome),<br>nrfE (putative cytochrome c-type biogenesis protein NrfE),<br>nrfF (putative formate-dependent nitrite reductase complex subunit NrfF),<br>nrfG (putative formate-dependent nitrite reductase complex subunit NrfG),<br>sdhC (succinate:quinone oxidoreductase, membrane protein SdhC),<br>sdhD (succinate:quinone oxidoreductase, membrane protein SdhD),<br>torC (cytochrome *c* menaquinol dehydrogenase TorC),<br>yceJ (putative cytochrome b561)<br><br>Thioredoxin, glutaredoxin (7)<br>grxA (oxidized glutaredoxin 1)<br>grxB (oxidized glutaredoxin 2),<br>grxC (oxidized glutaredoxin 3),<br>grxD (glutaredoxin 4),<br>nrdH (glutaredoxin-like protein),<br>trxA (oxidized thioredoxin),<br>trxC (oxidized thioredoxin 2)
Lipopolysaccharide (56)	asmA (putative assembly protein AsmA)<br>kdsC (3-deoxy-D-*manno*-octulosonate 8-phosphate phosphatase KdsC)<br>lapA (lipopolysaccharide assembly protein A)<br>lapB (lipopolysaccharide assembly protein B)

	lptD (lipopolysaccharide assembly protein LptD)<br>lptE (lipopolysaccharide assembly protein LptE)<br><br>core region (22)<br><br>eptB (Kdo2-lipid A phosphoethanolamine 7''-transferase),<br>gmhB (D-*glycero*-β-D-*manno*-heptose-1,7-bisphosphate 7-phosphatase),<br>gutQ (D-arabinose 5-phosphate isomerase GutQ),<br>kdsA (3-deoxy-D-*manno*-octulosonate 8-phosphate synthase),<br>kdsB (3-deoxy-*manno*-octulosonate cytidylyltransferase),<br>kdsD (D-arabinose 5-phosphate isomerase KdsD),<br>lpcA (D-sedoheptulose 7-phosphate isomerase),<br>lpxK (tetraacyldisaccharide 4'-kinase),<br>rfaD (ADP-L-glycero-D-mannoheptose 6-epimerase),<br>rfaE (fused heptose 7-phosphate kinase/heptose 1-phosphate adenyltransferase),<br>rfaH (transcription antiterminator RfaH),<br>waaA (KDO transferase),<br>waaC (ADP-heptose:LPS heptosyltransferase I),<br>waaF (ADP-heptose—LPS heptosyltransferase 2),<br>waaG (lipopolysaccharide glucosyltransferase I),<br>waaL (O-antigen ligase),<br>waaP (lipopolysaccharide core heptose (I) kinase),<br>waaQ (lipopolysaccharide core heptosyltransferase III),<br>waaS (lipopolysaccharide core biosynthesis protein),<br>waaU (lipopolysaccharide heptosyltransferase),<br>waaY (lipopolysaccharide core heptose (II) kinase),<br>waaZ (lipopolysaccharide core biosynthesis protein WaaZ)<br><br>lipid A (15),<br><br>arnA (fused UDP-4-amino-4-deoxy-L-arabinose formyltransferase/UDP-glucuronate dehydrogenase),<br>arnB (UDP-4-amino-4-deoxy-L-arabinose aminotransferase),<br>arnC (undecaprenyl-phosphate 4-deoxy-4-formamido-L-arabinose transferase),<br>arnT (lipid IV_A 4-amino-4-deoxy-L-arabinosyltransferase),<br>lpxA (UDP-N-acetylglucosamine acyltransferase),<br>lpxB (lipid A disaccharide synthase),<br>lpxC (UDP-3-*O*-acyl-*N*-acetylglucosamine deacetylase),<br>lpxD (UDP-3-*O*-(3-hydroxymyristoyl)glucosamine *N*-acyltransferase),<br>lpxH (UDP-2,3-diacylglucosamine diphosphatase),<br>lpxK (tetraacyldisaccharide 4'-kinase),<br>lpxL (lauroyl acyltransferase),<br>lpxM (myristoyl-acyl carrier protein-dependent acyltransferase),<br>lpxP (palmitoleoyl acyltransferase),

	pagP (Lipid IV_A palmitoyltransferase),<br>waaA (KDO transferase)<br><br>O antigen (14)<br><br>glf (UDP-galactopyranose mutase),<br>glmM (phosphoglucosamine mutase),<br>glmS (L-glutamine—D-fructose-6-phosphate aminotransferase),<br>glmU (fused N-acetylglucosamine-1-phosphate uridyltransferase and glucosamine-1-phosphate acetyltransferase),<br>rfbA (dTDP-glucose pyrophosphorylase),<br>rfbB (dTDP-glucose 4,6-dehydratase 1),<br>rfbC (dTDP-4-dehydrorhamnose 3,5-epimerase),<br>rfbD (dTDP-4-dehydrorhamnose reductase),<br>rfbX (polyisoprenol-linked *O*-antigen repeat unit flippase),<br>rffG (dTDP-glucose 4,6-dehydratase 2),<br>rffH (dTDP-glucose pyrophosphorylase 2),<br>wbbH (putative O-antigen polymerase),<br>wbbI (β-1,6-galactofuranosyltransferase),<br>wzzB (regulator of length of O-antigen component of lipopolysaccharide chains)
Lipoprotein (2)	lnt (apolipoprotein N-acyltransferase),<br>ybaY (PF09619 family lipoprotein YbaY)
Murein (peptidoglycan) (53)	amiA (*N*-acetylmuramoyl-L-alanine amidase A),<br>amiB (*N*-acetylmuramoyl-L-alanine amidase B),<br>amiC (*N*-acetylmuramoyl-L-alanine amidase C),<br>ampC (β-lactamase),<br>dacA (D-alanyl-D-alanine carboxypeptidase DacA),<br>dacB (peptidoglycan DD-endopeptidase DacB),<br>dacC (D-alanyl-D-alanine carboxypeptidase DacC),<br>dacD (D-alanyl-D-alanine carboxypeptidase DacD),<br>ddlA (D-alanine—D-alanine ligase A),<br>ddlB (D-alanine—D-alanine ligase B),<br>ddpX (D-alanyl-D-alanine dipeptidase),<br>elyC (envelope biogenesis factor),<br>ftsI (peptidoglycan DD-transpeptidase FtsI),<br>glmM (phosphoglucosamine mutase),<br>glmS (L-glutamine—D-fructose-6-phosphate aminotransferase),<br>glmU (fused N-acetylglucosamine-1-phosphate uridyltransferase and glucosamine-1-phosphate acetyltransferase),<br>ispU (undecaprenyl diphosphate synthase),<br>ldtA (L,D-transpeptidase ErfK),<br>ldtB (L,D-transpeptidase YbiS),<br>ldtC (L,D-transpeptidase YcfS),<br>ldtD (L,D-transpeptidase LdtD),<br>ldtE (L,D-transpeptidase LdtE),<br>lpoA (outer membrane lipoprotein - activator of MrcA activity),<br>lpoB (outer membrane lipoprotein - activator of MrcB activity),<br>lpp (murein lipoprotein),<br>mepA (peptidoglycan DD-endopeptidase/peptidoglycan LD-endopeptidase),<br>mepH (peptidoglycan DD-endopeptidase MepH),

	mepM (peptidoglycan DD-endopeptidase MepM),<br>mepS (peptidoglycan DD-endopeptidase/peptidoglycan LD-carboxypeptidase),<br>mipA (scaffolding protein that interacts with murein polymerase and murein hydrolase),<br>mltG (endolytic murein transglycosylase),<br>mraY (phospho-*N*-acetylmuramoyl-pentapeptide-transferase),<br>mrcA (peptidoglycan glycosyltransferase / peptidoglycan DD-transpeptidase MrcA),<br>mrcB (peptidoglycan glycosyltransferase / peptidoglycan DD-transpeptidase MrcB),<br>mrdA (peptidoglycan DD-transpeptidase MrdA),<br>mtgA (peptidoglycan glycosyltransferase MtgA),<br>murA (UDP-*N*-acetylglucosamine 1-carboxyvinyltransferase),<br>murB (UDP-*N*-acetylenolpyruvoylglucosamine reductase),<br>murC (UDP-*N*-acetylmuramate—L-alanine ligase),<br>murD (UDP-*N*-acetylmuramoyl-L-alanine—D-glutamate ligase),<br>murE (UDP-*N*-acetylmuramoyl-L-alanyl-D-glutamate—2,6-diaminopimelate ligase),<br>murF (D-alanyl-D-alanine-adding enzyme),<br>murG (N-acetylglucosaminyl transferase),<br>murI (glutamate racemase),<br>pbpC (peptidoglycan glycosyltransferase PbpC),<br>pbpG (peptidoglycan DD-endopeptidase PbpG),<br>pgpB (phosphatidylglycerophosphatase B),<br>prc (tail-specific protease),<br>ybjG (undecaprenyl pyrophosphate phosphatase)
Osmoregulated periplasmic glucan (3)	opgB (phosphoglycerol transferase II),<br>opgC (protein required for succinyl modification of osmoregulated periplasmic glucans),<br>opgG (osmoregulated periplasmic glucans (OPGs) biosynthesis protein G)

The MultiFun ontology assigns genes to ontology classes based on the function of the gene product.
In red are shown the non-viable mutant strains
In yellow selected knock out mutants
