# Supplementary material for: A genetically engineered Escherichia coli strain overexpressing the nitroreductase NfsB is capable of producing the herbicide D-DIBOA with 100% molar yield
Source: Microb Cell Fact. 2019 May 20;18:86. doi: 10.1186/s12934-019-1135-8 (PMC6526606; doi:10.1186/s12934-019-1135-8)
Supplement: Supplementary file 3 — Additional file 3. List of the selected superpathways and genes involved in carbon and energy metabolism. [file 12934_2019_1135_MOESM3_ESM.pdf]

Additional file 3. List of the selected superpathways and genes involved in carbon and energy metabolism.

**Superpathways (MultiFun Ontology: Metabolism, Child classes)**	**Subclases (number of genes)**	**Selected knock out mutant**
Carbon utilization (399)	Amines (38)<br>Amino acids (54)<br>Other compounds (1)	
	Fatty acids (31)	fadR (DNA-binding transcriptional dual regulator FadR)
	Carbon compounds (269)	pfkA (6-phosphofructokinase I)<br>pfkB (6-phosphofructokinase II)
Central intermediary metabolism (278)	Sugar nucleotide biosynthesis, conversions (12)	sthA (soluble pyridine nucleotide transhydrogenase)
Energy metabolism, carbón (203)	Aerobic respiration (44)	nuoA (NADH:quinone oxidoreductase subunit A),<br>nuoB (NADH:quinone oxidoreductase subunit B),<br>nuoE (NADH:quinone oxidoreductase subunit E),<br>nuoF (NADH:quinone oxidoreductase subunit F),<br>nuoG (NADH:quinone oxidoreductase subunit G),<br>nuoN (NADH:quinone oxidoreductase subunit N),
	Glycolysis (13)	pgi (glucose-6-phosphate isomerase),
Energy production/transport (96)	Electron acceptors (31)	narG (nitrate reductase A subunit α)
	Electron carriers (15)<br>Electron donors (43)	
