# Supplementary material for: A genetically engineered Escherichia coli strain overexpressing the nitroreductase NfsB is capable of producing the herbicide D-DIBOA with 100% molar yield
Source: Microb Cell Fact. 2019 May 20;18:86. doi: 10.1186/s12934-019-1135-8 (PMC6526606; doi:10.1186/s12934-019-1135-8)

Additional file 4. Biotransformation yields in the biocatalysis carried out by Δ*lapA,* Δ*fliQ,* Δ*nuoG* and Δ*fadR* single mutant strains, Δ*lapA*Δ*fliQ* double mutant strain and wild type strain at 4, 8, 18 and 24 h of experiment after precursor addition.

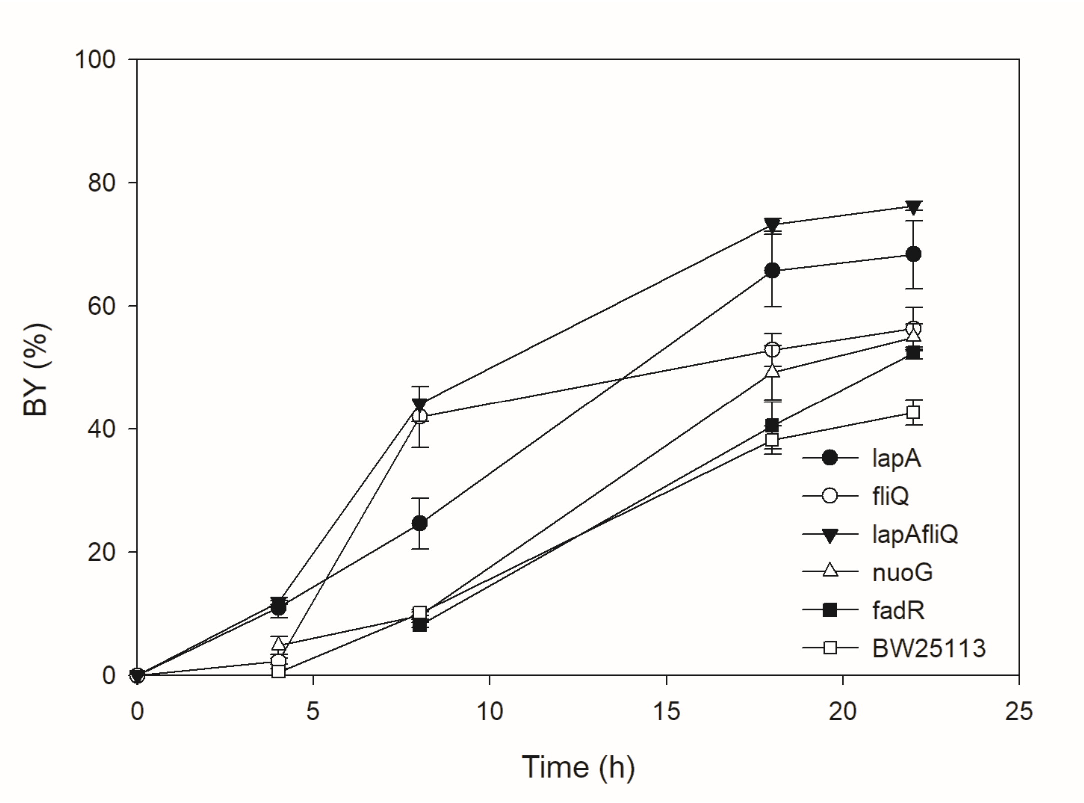

Supplement: Supplementary file 4 — Additional file 4. Biotransformation yields in the biocatalysis carried out by ∆lapA, ∆fliQ, ∆nuoG and ∆fadR single mutant strains, ∆lapA∆fliQ double mutant strain and wild type strain at 4, 8, 18 and 24 h of experiment after precursor addition. [file 12934_2019_1135_MOESM4_ESM.pdf]
