# Supplementary material for: A genetically engineered Escherichia coli strain overexpressing the nitroreductase NfsB is capable of producing the herbicide D-DIBOA with 100% molar yield
Source: Microb Cell Fact. 2019 May 20;18:86. doi: 10.1186/s12934-019-1135-8 (PMC6526606; doi:10.1186/s12934-019-1135-8)

Additional file 5. Growth curves for: (a) Biotransformations with the Δ*lapA,* Δ*fliQ* and Δ*lapA*Δ*fliQ* strains described in Fig. 3 c and d. (b) Biotransformation with the Δ*lapA*Δ*fliQ* double mutant in the multiple loads of precursor experiments described in Fig. 6

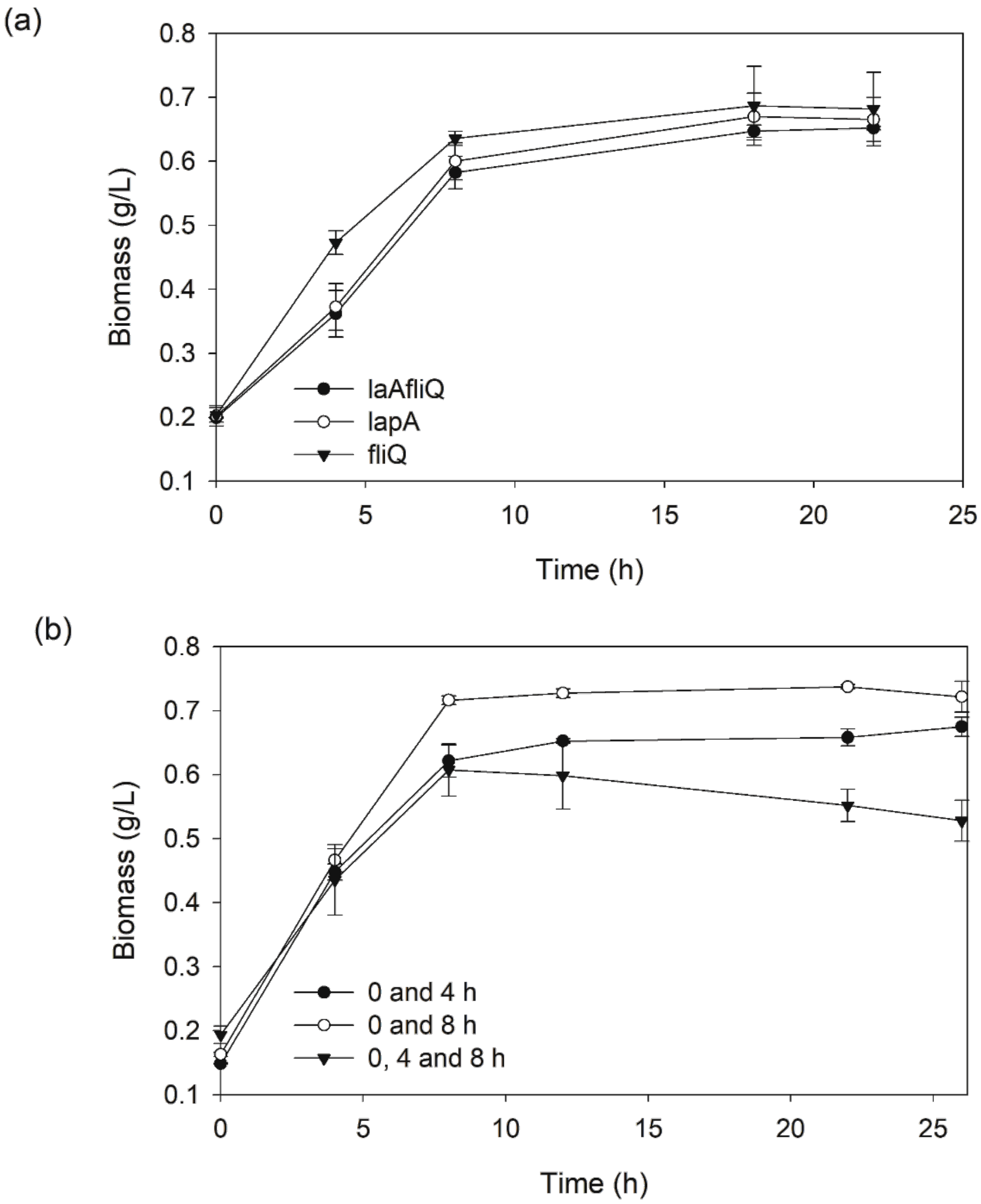

Supplement: Supplementary file 5 — Additional file 5. Growth curves for: (a) Biotransformations with the ΔlapA, ΔfliQ and ΔlapAΔfliQ strains described in Fig. 3c and d. (b) Biotransformation with the ΔlapAΔfliQ double mutant in the multiple loads of precursor experiments described in Fig. 6. [file 12934_2019_1135_MOESM5_ESM.pdf]
